# Commentary on: Awake Plastic Surgery Procedures: The Use of a Sufentanil Sublingual Tablet to Improve Patient Experience

**DOI:** 10.1093/asjof/ojac003

**Published:** 2022-02-03

**Authors:** Mikaela L Kislevitz, Jayne Coleman

**Affiliations:** Department of Plastic and Reconstructive Surgery, University of Texas Southwestern Medical Center, Dallas, TX, USA; Department of Plastic and Reconstructive Surgery, University of Texas Southwestern Medical Center, Dallas, TX, USA

This article^1^ highlights the benefits of utilizing pain medications to allow for outpatient, in-office awake surgical plastic surgery operations. However, using sufentanil sublingual tablets may have more risks than the authors addressed.

Sufentanil is a fentanyl analog with an extremely high therapeutic index. It is 5 to 10 times more potent than fentanyl. In the article, the authors stated that no vital sign instability or oxygen desaturation was observed. Although it has been reported in some studies to cause less respiratory depression, it still contains the black box warning for respiratory depression. Sufentanil is more lipophilic than fentanyl with a fast onset, and it is advised to be used in a medically supervised setting if it should lead to respiratory depression.^2^

In the era of opioid crises, oral sufentanil has a strong abuse potential. Sufentanil is not a drug that can be obtained from a local pharmacy and must be administered under medically supervised healthcare settings that include hospitals, surgical centers, and emergency rooms.^3^ In addition, sufentanil can become more potent with other drugs including cytochrome P450 inhibitors and with high levels can be fatal. Patients with comorbidities including chronic obstructive pulmonary disease may be more vulnerable to respiratory depression.^3^ It was not clearly outlined by the authors of this study where exactly these awake plastic surgery procedures occurred and under what supervision. The article mentioned that supplemental oxygen, bag valve mask, and naloxone were available to treat respiratory depression. Vital signs (pulse, respiratory rate, blood pressure, and oxygen saturation) were recorded before and after the procedure.

Although this article introduced the benefits of minimizing anesthetics and performing more procedures in an outpatient setting, given the black box warning, we still feel that this drug may be best to be monitored by anesthesiologists in a medically supervised healthcare setting.
